# Associations between Nurse Staffing Levels, Patient Experience, and Hospital Rating

**DOI:** 10.3390/healthcare9040387

**Published:** 2021-04-01

**Authors:** Kyung Jin Hong, Sung-Hyun Cho

**Affiliations:** 1 Department of Nursing, Semyung University, Jecheon 27136, Korea; kjhong@semyung.ac.kr; 2 College of Nursing, Research Institute of Nursing Science, Seoul National University, Seoul 03080, Korea

**Keywords:** nurse, nurse staffing, patient satisfaction, patient experience, hospital rating

## Abstract

The current study aimed to examine patient experience scores and differences in the scores based on the region and nurse staffing level of hospitals as well as to verify the effect of nurse-related patient experience scores on the overall rating of hospitals. Secondary data from the second Korean Patient Experience Survey—conducted using the cross-sectional design method—were analyzed, and 146 hospitals were included. Patient experience scores included six dimensions, and hospitals were categorized as: tertiary or general hospitals based on their type; capital and non-capital region hospitals based on regions; and beds-nurse or patients-nurse ratios were used based on nurse staffing levels. Pearson’s correlation, simple regression, and multiple regression analysis methods were used. Among the six patient experience dimensions, the nurse-related patient experience score of 86.0 was the highest, whereas patient rights score of 78.4 was lowest. Moreover, the patient experience score for general hospitals with low nurse staffing grade was low, and the nurse experience score affected the overall hospital rating in general hospitals (*p* = 0.040). Policies to improve nurse staffing level are required to provide high-quality nursing care focused on communication with patients, which can enhance patient experience and satisfaction.

## 1. Introduction

A patient is regarded as a consumer who selects and consumes healthcare services rather than a recipient of the same [1]. Therefore, policy makers and hospital administrators consider patient experience as an important factor to assess the quality of the healthcare system [2]. Patient experience is defined as the sum of all interactions shaped by an organization’s culture that influence patient perceptions across the continuum of care [3]. In the United States, the Hospital Valued-Based Purchasing Program adjusts hospital payments based on a hospital’s performance including patient experiences [4]. Patient experience has also been associated with patient outcomes [5,6]. Further, surveys regarding patient experience and the publication of their results helps patients choose healthcare facilities [7], and healthcare establishments can also use this feedback to improve patient experience [8]. In South Korea, based on the recommendation of the OECD (Organization for Economic Cooperation and Development) to improve patient-centered care [9], the Health Insurance Review and Assessment Service (HIRA) conducted surveys to measure inpatient experience, with the second survey being conducted in 2019 [10]. The dimensions and question items were not changed from the first survey, but there were only a few corrections of vocabulary and scale.

Including nurses in the evaluation of the patient experience is very important because nurses are the patients’ bedside staff and the most visible health professionals who patients encounter during a hospital stay [11]. Moreover, nurses provide information and educate patients from the beginning of their stay in the hospital until discharge. Therefore, sufficient nurse staffing is very important for improving patient experience. Nurse staffing levels are likely to affect the nurses’ ability to improve patient experience, because nurses who work on units that are understaffed or do not have the adequate skill levels are likely to have less time to respond to patient requests and educate them [2,12]. Previous studies found that inadequate staffing levels result in patient mortality, failure-to-rescue rates, nurse burnout and dissatisfaction, and missed nursing care [13,14,15]. Therefore, nurse staffing levels could be related to the patient experience related to nurses’ care. It was found that nurse staffing levels differed by type and region of hospital because newly graduated nursing students prefer to be employed in large hospitals in the capital region considering wage and treatment welfare [16,17]. This means that since there could be differences in nurse staffing grades and patient experience scores according to type and region, the variables should be considered to explain patient experience. However, previous studies only explained the relationship between nurse staffing level and patient experience, without sufficiently considering hospital characteristics such as region and type [18,19]. Therefore, this study examined whether patient experience scores would differ according to hospital characteristics and nurse staffing levels.

Nursing care includes having communication with patients and caregivers, focusing on the process of medication and treatment, and providing information on admission and discharge. Patient experience related to nursing care affects the overall hospital rating, and the results can be used as evidence to discuss the need for high-quality nursing care to improve patient experience and satisfaction. Although previous studies reported the effects of nurse-related patient experiences on the overall hospital rating, the differences in nurse staffing grade were not examined according to hospital characteristics [20]. Therefore, this study analyzed whether a better nurse-related patient experience would lead to better scores in the overall hospital rating, taking into account hospital characteristics such as type, region of hospitals, and nurse staffing level. The necessity of improving the nurse staffing level and the quality of nursing care according to the hospital type or region was explained by confirming the nurse staffing level according to the characteristics of the hospitals and analyzing the overall hospital evaluation according to the patients’ nurse-related experience.

This study aimed to (1) examine patient experiences according to the hospitals’ characteristics, including nurse staffing levels, and to (2) analyze the effects of nurse-related patient experiences on the overall hospital rating.

## 2. Materials and Methods

### 2.1. Design

This study was based on secondary data analysis, and used public data was obtained from the Patient Experience Survey in South Korea, which was conducted in 2019 by the HIRA. The survey used the telephonic interview method and cross-sectional design.

### 2.2. Sample and Setting

The ‘patient experience survey’ included 154 tertiary and general hospitals with 300 or more beds and surveyed patients who had been hospitalized for one day or more at these hospitals within 2 to 56 days after discharge. A total of 23,924 patients who were ever hospitalized in the 154 hospitals responded. The survey was conducted via telephonic interviews, and data were collected from May to November 2019. The hospital name and patient experience score with six dimensions were published on the HIRA website’s homepage [21]. We downloaded the list of evaluated hospitals and searched and recorded the detailed patient experience scores and nurse staffing levels for the third quarter of 2019 from the same webpage. The inclusion criteria for the analysis comprised answering every item related to nurse staffing levels and patient experience scores. The final sample included 146 tertiary and general hospitals.

### 2.3. Instruments

#### 2.3.1. Hospital Characteristics

Items related to the characteristics of hospitals were the type and region of hospital. Based on type, the hospitals were divided into tertiary and general hospitals, and based on region, they were divided into capital-region (Seoul, Incheon, and Gyeonggi) and non-capital region hospitals. The nurse staffing grade used the data published on the HIRA website. In South Korea, the number of nurses and beds (or patients) to calculate the nurse staffing grade should be notified to HIRA on a quarterly basis to adjust differentiated nursing fees. HIRA calculates the health insurance fee schedule based on the nurse staffing grade. The nurse-bed ratio was applied to tertiary hospitals or capital region general hospitals to determine the nurse staffing grade. Moreover, the nurse-patient ratio was applied to general hospitals in non-capital regions to calculate the nurse staffing grade that reflected occupancy rates [22]. The nurse staffing grade for tertiary hospitals was categorized to 1–6: grade 1 denoted a score lower than 2.0, grade 2, 2.0–<2.5, grade 3, 2.5–<3.0, and grade 6, ≥4.0. The nurse staffing grade of general hospitals was categorized across to 1–7, with grade 1 being lower than 2.5, grade 2, 2.5–<3.0, grade 3, 3.0–<3.5, grade 4, 3.5–<4.0, grade 5, 4.0–<4.5, grade 6, 4.5–<6.0, and grade 7, ≥6.0. Therefore, grade 1 indicates the highest staffing, whereas grade 6 in tertiary hospitals and grade 7 in general hospitals are the lowest staffing. Nurse staffing grades 4–7 of general hospitals were considered as one group because they were a small sample.

#### 2.3.2. Patient Experience Score

The patient experience score had six dimensions: nurse-related factors, physician-related factors, medication and treatment, hospital environment, patient rights, and overall hospital rating. The nurse-related factors comprised four items: courtesy/politeness, ability to listen carefully, explanation regarding hospitalization, and effort taken to handle patient requirements; physician-related factors comprised four items: courtesy/politeness, ability to listen carefully, opportunity to meet with a physician, and providing information about the physician’s rounds schedule. Medication and treatment comprised five items: e.g., effort taken to control pain, providing information regarding precautions and plan of further medication and treatment after discharge. Hospital environment comprised two items: cleanliness and safe environment. Patient rights included four items: e.g., fair treatment and ease of discussing dissatisfaction. Each item was rated 1–4, except for one item (providing information regarding precautions and plan of further medication and treatment after discharge was rated 1–2). A higher score means that patients experienced a higher quality of care. Finally, the overall hospital rating included two items: overall evaluation and willingness to recommend the hospital to friends and families and was rated 0–10. All items were normalized to 0–100; for example, 1–4 was normalized to 0, 33, 66, and 100, and the scores of the six dimensions (range: 0–100) were published.

### 2.4. Data Analysis

The data were analyzed using SPSS version 23.0 (IBM Corp., Armonk, NY, USA). A descriptive analysis of the six dimensions of patient experience was performed, and Pearson’s correlation was used to examine the relationship between the six dimensions. The factors influencing the overall hospital rating were analyzed using simple and multiple regression analyses. After examining the normality of the variables, the simple regression method was used to compare each patient experience score according to hospital characteristics, including nurse staffing grades, and examine the effects of each patient experience score on the overall hospital rating. Multiple regression was used to determine whether nurse-related patient experience scores had an effect on the overall hospital rating, over and above the influence exerted by other patient experience dimensions. A 95% confidence interval was established, considering statistically significant results at *p* < 0.05.

### 2.5. Ethical Consideration

Because this study was based on secondary data analysis, the aims and methods of the study were reviewed and exempted by the Institutional Review Board of the first author’s institution (IRB No. SMU-EX-2021-01-001). The ‘Patient Experience Survey’ was conducted by KIHA and was mandatory for tertiary and general hospitals with more than 300 beds in South Korea. The data of individual patients were not disclosed, and the included data did not contain any information that could identify the individual participants.

## 3. Results

### 3.1. Descriptive Results of Patient Experience Scores Based on Hospital Characteristics

The descriptive results of patient experience scores based on hospital characteristics are shown in Table 1. Among the 146 hospitals, there were 42 tertiary—21 capital and 21 non-capital region—and 104 general hospitals—47 capital and 57 non-capital region. The nurse staffing grade of tertiary hospitals was grade 1 for 15 and grade 2 for 27 hospitals; whereas for general hospitals, 55 hospitals secured grade 1, 23 secured grade 2, 10 secured grade 3, and 16 secured grade 4–7. The highest score among the six dimensions of patient experience was for nurse-related factors, with a mean of 86.0, whereas the lowest was for patient rights, with a mean of 78.4. The mean overall hospital rating score was 82.1. In tertiary hospitals, the nurse experience score was 87.5, and the score for the capital region was 88.2, which was higher than that for the non-capital region (86.8). The nurse experience score for the tertiary hospitals with nurse staffing 1 was 87.7 and that of hospitals with nurse staffing 2 was 87.4. The overall hospital rating score was 85.2 for the capital region, higher than that for the non-capital region (83.3), 85.4 for tertiary hospitals with nurse staffing grade 1, and 83.7 for those with nurse staffing grade 2. In general hospitals, the nurse-related patient experience score was 85.4, and the score for the capital region (86.3) was higher than that of the non-capital region (84.6). The nurse-related patient experience score for grade 1 hospitals was 86.8, higher than that of general hospitals with lower nurse staffing grade; the nurse-related patient experience score of general hospitals with grade 4–7 was the lowest (82.5). For all six dimensions, the score of patient experience for general hospitals with nurse staffing grade 1 was the highest.

### 3.2. Correlation among Patient Experience Dimensions

Table 2 shows the Pearson correlation coefficients (*p*-values) of patient experience dimensions. The nurse-related patient experience score was correlated with the other five patient experience dimensions for both types of hospitals. Moreover, the overall hospital rating score was correlated with the other five dimensions. The coefficients between nurse patient scores and overall hospital ratings were 0.773 for tertiary hospitals (<0.001), and 0.797 for general hospitals (<0.001).

### 3.3. Simple Regression Results for Patient Experience Dimensions

Table 3 shows the results of a simple regression for patient experience dimensions. Nurse staffing grade 1 was used as a reference group to verify the differences in patient experience scores according to nurse staffing grades, and compare the patient experience scores of healthcare facilities with lower nurse staffing grades as compared to those with the highest nurse staffing grades. In tertiary hospitals, hospital environment score for hospitals in the capital region were significantly higher than that of hospitals in the non-capital region (*p* = 0.014). The differences in patient experience score according to nurse staffing grade were not significant. In general hospitals, nurse-related factors, medication and treatment, hospital environment, and patient rights scores of hospitals with nurse staffing grade 1 were higher than those of hospitals with nurse staffing grade 2, 3, and 4–7. The overall hospital rating of hospitals with nurse staffing grade 1 was higher than that of hospitals with nurse staffing grade 3 and 4–7 (*p* < 0.001).

### 3.4. Effects of Each Patient Experience Dimension on Overall Hospital Rating

Table 4 shows the simple and multiple regression results for the overall hospital rating. In the simple regression analysis, the five dimensions of patient experience, including nurse-related patient experience score affected the overall hospital rating in both tertiary and general hospitals. The coefficient of nurse-related patient experience score to the overall hospital rating was 1.01 (*p* < 0.001) for tertiary hospitals and 1.14 (*p* < 0.001) for general hospitals. In the multiple regression, the nurse-related patient experience did not affect the overall hospital rating, whereas the hospital environment affected the rating (*p* < 0.001) for tertiary hospitals. The result of the multiple regression for general hospitals indicated that the nurse-related patient experience (*p* = 0.040), medication and treatment (*p* < 0.001), and hospital environment (*p* < 0.001) affected the overall hospital rating.

## 4. Discussion

The current study was conducted to examine patient experiences according to hospitals’ characteristics, including nurse staffing levels and to analyze the effects of nurse-related patient experiences on the overall hospital rating. It was found that general hospitals with nurse staffing grades below grade 2 showed lower patient experience scores than those of general hospitals with nurse staffing grade 1, and that the score of nurse-related factors affected the overall hospital rating.

For the six dimensions of patient experience, the means ranged from 78.4 to 86.0, and differences among dimensions were relatively small. It showed that the overall quality of care of hospitals that performed the survey was evenly high. However, tertiary hospitals performed slightly better than general hospitals in all categories, as shown in a previous study [18]. This is because tertiary hospitals are required to provide medical services for severe diseases, and, therefore, high-risk patients, which is why they might have well-designed systems for the processes of various prescriptions, preparations, medications, and treatments [18]. The score for nurse-related factors was the highest (86.0). Nurses are important healthcare workers who support the patient from admission to the discharge processes and frequently have communication with patients. From the results of the patient experience survey, it can be seen that nurses provided moderate or high-quality nursing care, involving a polite attitude and sufficient effort to handle patient requirements. Among the six patient experience dimensions, for both tertiary and general hospitals, the score was the lowest for patient rights (78.4), followed by physician-related factors (81.4). The results were different from the previous survey conducted in the U.S., which showed the highest scores for communication with physicians [23]. In the report analyzed, the detailed result of all items revealed that patients were dissatisfied with the difficulty in filing complaints and meeting physicians because of not being given information about the physicians’ rounds schedule [24]. The results revealed that it is necessary to increase the opportunities for patients to meet physicians by providing them with information about the physicians’ rounds schedule and create an atmosphere where it is easy to raise complaints. Moreover, the score of patient rights was the lowest—consistent with a previous study—which explained that it is still cumbersome to file complaints due to concerns over unfair treatment of medical services, despite improvement in the awareness of patients’ rights and demand for medical services [25]. Hospitals should make efforts to ease the redressal seeking process for patients.

The nurse-related patient experience scores of tertiary hospitals did not differ by region and nurse staffing level of hospitals. Because the variance in nurse staffing grade of tertiary hospitals was fairly small (1 or 2), the impact of nurse staffing on patient experience may not be found. In a previous study conducted in South Korea, the level of nurse staffing in tertiary hospitals improved after the implementation of the law regarding differentiated nursing fees [17,26], and the gap in nurse staffing grade between tertiary hospitals in capital and non-capital regions was narrowed. On the other hand, tertiary hospitals in the non-capital region showed a lower score with respect to the hospital environment than that of hospitals in the capital region; hence, there may be geographic differences that affect the cleanliness and safety of the hospital environment. Further studies are needed to verify these differences by adjusting various hospital and patient characteristics to enhance infection control and patient outcomes in non-capital region hospitals.

Nurse staffing was found to be a factor affecting patient experience in general hospitals, and this result was in line with previous studies [27,28]. In previous studies, patient experience scores were related to nurse staffing, because in a work environment with an adequate number of well-trained nurses, nurses are able to provide critical surveillance of their patients, instruct patients on medication and discharge, and provide critical bedside care [29,30]. However, insufficient nurse staffing was related to missed care (left undone, omission nursing care), and the most frequent aspects of missed care included lack of communication such as comforting/talking to patients, providing emotional, psychological support, and educating patients [31]. Patients’ perceptions of hospital care were strongly associated with missing nursing care, which in turn was related to poor registered nurse staffing [32]. Better nurse staffing levels could affect the quality of nursing care, including patient-centered care, through sufficient communication, teaching, and explanation regarding the medication and treatment process. In South Korea, there are legal standards for nurse staffing; however, many medical institutions do not meet the legally mandated minimum staffing level [33]. Policy action of the government is required in the case of medical institutions so that they meet nurse staffing standards.

Moreover, the nurse-related patient experience affects the overall hospital rating in general hospitals, and the necessity to include nurses’ care for evaluating patient experience was supported. A previous study conducted in Korea also showed the positive affect of nurse staffing level on the overall evaluation of hospitals. Further, it was discussed that the nurse staffing level is an important factor influencing the patient experience evaluation and can play an important factor in patients’ hospital selection [34]. Because nurse staffing levels in general hospitals were lower compared to those in tertiary hospitals, the effect of nurses’ scores on the overall hospital rating would be significant only in general hospitals. A patient-reported missed care mediated the relationship between staffing adequacy and the overall hospital rating, therefore, the necessity of staffing adequacy to improve patient experience was identified [35]. The items of the patient experience survey focused on sufficient communication between the patient and the nursing staff in the dimensions of nurse-related factors, medication and treatment, and patient rights, which would be related to the patient experience score. Nurse communication is integral to patients’ perception of their overall care, such as pain management and communication regarding medication, and has a direct and positive impact on the overall hospital rating [36]. Therefore, high-quality nursing care focusing on nurse communication by ensuring adequate nurse staffing can improve patient experience and satisfaction.

The current study was conducted to evaluate the patient experience score and differences in patient experience based on hospital characteristics such as region and nurse staffing level as well as to verify the effect of nurse experience on the overall hospital rating. Improving nurse staffing levels is required to provide patient-centered care and sufficient communication. Policies to improve nurse staffing levels for providing high-quality nursing care and sufficient communication as well as enhancing efforts to respond to patient requests or complaints are required. It is necessary to include patients in the medication and treatment processes and enhancing patient outcomes and satisfaction.

The current study had some limitations. Exploring each item of the patient experience survey in detail was not possible because the institutions conducting the “patient experience survey” only disclosed the mean scores of the six dimensions, and not the specific mean scores for each of the items. Unobserved hospital characteristics may not be included in the study, and the data might not be relevant to all hospitals in South Korea. Future studies should include unobserved hospital factors, such as the leadership of managers, work environment, and reward from work, and each item of the patient experience score included in the six dimensions should be analyzed to explain the detailed patient experiences. Moreover, health care facilities of various sizes should be included in the survey to generalize our findings, and the changes in patient experience scores should be explained by a longitudinal study.

## 5. Conclusions

This study examined the patient experience score and differences in patient experience based on the region and nurse staffing levels of hospitals. Further, it verified the effect of nurse patient experience score on the overall hospital rating in general hospitals. An adequate nurse staffing level enables nurses to provide sufficient and clear explanations about the process of medication and treatment, which can improve patient satisfaction of nursing care. Hospital managers should consider patient experience as an important factor affecting patient-centered care and nurses as pivotal healthcare providers who can enhance the patient experience.

## Figures and Tables

**Table 1:** Descriptive results of patient experience scores based on hospital characteristics.

		Patient Experience, M ± SD					
	n (%)	Nurses	Physicians	Medication and Treatment	Hospital Environment	Patient Rights	Overall Hospital Rating
All hospitals	146	86.0 ± 3.0	81.4 ± 2.6	82.7 ± 2.8	82.2 ± 4.7	78.4 ± 3.1	82.1 ± 4.3
Tertiary hospitals	42	87.5 ± 2.5	81.8 ± 2.8	84.0 ± 2.6	84.3± 3.8	79.6 ± 3.3	84.3 ± 3.3
Region							
Capital	21 (50.0)	88.2 ± 2.5	82.1 ± 2.9	84.4 ± 2.7	85.8 ± 3.6	80.2 ± 3.5	85.2 ± 3.3
Non-capital	21 (50.0)	86.8 ± 2.4	81.4 ± 2.8	83.6 ± 2.5	82.9 ± 3.6	79.1 ± 3.1	83.3 ± 3.1
Nurse staffing							
Grade 1	15 (35.7)	87.7 ± 2.5	82.3 ± 2.8	84.6 ± 2.5	85.7 ± 4.1	80.3 ± 3.2	85.4 ± 3.0
Grade 2	27 (64.3)	87.4 ± 2.5	81.4 ± 2.8	83.7 ± 2.6	83.6 ± 3.5	79.2 ± 3.3	83.7 ± 3.3
General hospitals	104	85.4 ± 3.0	81.3 ± 2.6	82.2 ± 2.8	81.4 ± 4.7	78.0 ± 2.9	81.3 ± 4.3
Region							
Capital	47 (45.2)	86.3 ± 2.5	81.3 ± 2.5	82.5 ± 2.7	81.9 ± 4.7	78.2 ± 3.0	81.7 ± 4.5
Non-capital	57 (54.8)	84.6 ± 3.2	81.3 ± 2.7	81.9 ± 2.8	80.9 ± 4.7	77.8 ± 2.8	80.9 ± 4.2
Nurse staffing							
Grade 1	55 (52.9)	86.8 ± 2.1	81.9 ± 2.3	83.3 ± 2.3	83.7 ± 3.8	79.2 ± 2.7	82.9 ± 3.5
Grade 2	23 (22.1)	85.2 ± 2.4	80.8 ± 2.4	81.6 ± 2.4	80.5 ± 3.6	77.5 ± 2.7	81.0 ± 3.4
Grade 3	10 (9.6)	82.7 ± 3.3	80.3 ± 4.1	80.2 ± 3.7	77.3 ± 2.7	76.0 ± 3.0	77.5 ± 4.6
Grades 4-7	16 (15.4)	82.5 ± 3.2	80.6 ± 2.3	80.6 ± 2.9	77.6 ± 6.0	76.2 ± 3.0	78.6 ± 5.2

**Table 2:** Pearson correlation coefficients (*p*-values) among the patient experience dimensions (n = 146).

	Nurses	Physicians	Medication and Treatment	Hospital Environment	Patient Rights
Tertiary hospitals					
Nurses	1.00				
Physicians	0.771 (<0.001)	1.00			
Medication and treatment	0.897 (<0.001)	0.842 (<0.001)	1.00		
Hospital environment	0.655 (<0.001)	0.581 (0.001)	0.609 (<0.001)	1.00	
Patient rights	0.849 (<0.001)	0.839 (<0.001)	0.884 (<0.001)	0.677 (<0.001)	1.00
Overall hospital rating	0.773 (<0.001)	0.705 (<0.001)	0.776 (<0.001)	0.797 (<0.001)	0.754 (<0.001)
General hospitals					
Nurses	1.00				
Physicians	0.606 (<0.001)	1.00			
Medication and treatment	0.801 (<0.001)	0.819 (<0.001)	1.00		
Hospital environment	0.734 (<0.001)	0.491 (<0.001)	0.658 (<0.001)	1.00	
Patient rights	0.784 (<0.001)	0.703 (<0.001)	0.841 (<0.001)	0.717 (<0.001)	1.00
Overall hospital rating	0.797 (<0.001)	0.636 (<0.001)	0.828 (<0.001)	0.796 (<0.001)	0.741 (<0.001)

**Table 3:** Simple regression results for patient experience dimensions (n = 146).

	Nurses		Physicians		Medication and Treatment		Hospital Environment		Patient Rights		Overall Hospital Rating	
	Coeff.	95% CI (*p*)	Coeff.	95% CI (*p*)	Coeff.	95% CI (*p*)	Coeff.	95% CI (*p*)	Coeff.	95% CI (*p*)	Coeff.	95% CI (*p*)
Tertiary hospitals												
Capital region (vs. non-capital)	1.36	−0.16, 2.88 (0.078)	0.65	−1.12, 2.42 (0.462)	0.77	−0.84, 2.38 (0.341)	2.85	0.60, 5.10 (0.014)	1.06	−0.97, 3.10 (0.298)	1.88	−0.11, 3.87 (0.063)
Nurse staffing (vs. grade 1)												
grade 2	−0.25	−1.90, 1.39 (0.758)	−0.90	−2.74, 0.93 (0.326)	−0.89	−2.57, 0.79 (0.289)	−2.16	−4.60, 0.29 (0.082)	−1.09	−3.22, 1.04 (0.306)	−1.72	−3.82, 0.38 (0.105)
General hospitals												
Capital region (vs. non-capital)	1.65	0.51, 2.79 (0.005)	−0.05	−1.06, 0.96 (0.924)	0.64	−0.44, 1.72 (0.245)	0.98	−0.86, 2.82 (0.292)	0.45	−0.70, 1.60 (0.436)	0.80	−0.89, 2.49 (0.351)
Nurse staffing (vs. grade 1)												
Grade 2	−1.53	−2.76, −3.00 (0.015)	−1.03	−2.28, 0.21 (0.104)	−1.58	−2.83, −0.32 (0.014)	−3.07	−5.04, −1.10 (0.003)	−1.51	−2.84, −0.17 (0.027)	−1.85	−3.78, 0.08 (0.060)
Grade 3	−4.11	−5.82, −2.41 (<0.001)	−1.52	−3.25, 0.20 (0.083)	−3.01	−4.74, −1.28 (0.001)	−6.28	−9.01, −3.55 (<0.001)	−3.04	−4.88, −1.19 (0.002)	−5.34	−8.01, −2.67 (<0.001)
Grades 4–7	−4.25	−5.66, −2.84 (<0.001)	−1.28	−2.70, 0.21 (0.079)	−2.62	−4.05, −1.19 (<0.001)	−5.94	−8.20, −3.69 (<0.001)	−2.85	−4.37, −1.32 (<0.001)	−4.31	−6.52, −2.11 (<0.001)

CI: Confidence Interval.

**Table 4:** Effects of each patient experience dimension on overall hospital rating (n = 146).

	Simple Regression		Multiple Regression	
	Coefficient	95% CI (*p*)	Coefficient	95% CI (*p*)
Tertiary hospitals				
Nurses	1.01	0.75, 1.28 (<0.001)	0.17	−0.35, 0.68 (0.514)
Physicians	0.82	0.56, 1.09 (<0.001)	0.09	−0.29, 0.46 (0.646)
Medication and treatment	0.99	0.73, 1.24 (<0.001)	0.45	−0.13, 1.04 (0.126)
Hospital environment	0.68	0.52, 0.85 (<0.001)	0.43	0.24, 0.62 (<0.001)
Patient rights	0.76	0.55, 0.97 (<0.001)	−0.07	−0.47, 0.34 (0.735)
Adj. R^2^, F (*p*)			0.74, 24.61 (<0.001)	
General hospitals				
Nurses	1.14	0.97, 1.31 (<0.001)	0.25	0.01, 0.48 (0.040)
Physicians	1.06	0.81, 1.32 (<0.001)	−0.05	−0.31, 0.21 (0.697)
Medication and treatment	1.29	1.12, 1.46 (<0.001)	0.84	0.49, 1.18 (<0.001)
Hospital environment	0.73	0.62, 0.84 (<0.001)	0.38	0.26, 0.51 (<0.001)
Patient rights	1.10	0.90, 1.29 (<0.001)	−0.16	−0.42, 0.10 (0.221)
Adj. R^2^, F (*p*)			0.80, 84.66 (<0.001)	

CI: Confidence Interval.

## Data Availability

The data presented in this study are available upon request.
